# Alternating Current Electroosmotic Flow of Maxwell Fluid in a Parallel Plate Microchannel with Sinusoidal Roughness

**DOI:** 10.3390/mi15010004

**Published:** 2023-12-19

**Authors:** Long Chang, Guangpu Zhao, Mandula Buren, Yanjun Sun, Yongjun Jian

**Affiliations:** 1 School of Statistics and Mathematics, Inner Mongolia University of Finance and Economics, Hohhot 010070, China; suolunga@163.com (L.C.); sunyanjun.2006@163.com (Y.S.); 2 School of Mathematical Science, Inner Mongolia University, Hohhot 010021, China; 3 College of Sciences, Inner Mongolia University of Technology, Hohhot 010051, China; zhaoguangpu105@sina.com; 4 School of Mathematical Science, Inner Mongolia Normal University, Hohhot 010022, China; brmdllyc@163.com

**Keywords:** AC EOF, electric double layer (EDL), wall roughness, Maxwell fluid, microchannel, Deborah number

## Abstract

The EOF of a viscoelastic Maxwell fluid driven by an alternating pressure gradient and electric field in a parallel plate microchannel with sinusoidal roughness has been investigated within the Debye–Hückel approximation based on boundary perturbation expansion and separation of variables. Perturbation solutions were obtained for the potential distribution, the velocity and the mean velocity, and the relation between the mean velocity and the roughness. There are significant differences in the velocity amplitudes of the Newtonian and Maxwell fluids. It is shown here that the velocity distribution of the viscoelastic fluid is significantly affected by the roughness of the walls, which leads to the appearance of fluctuations in the fluid. Also, the velocity is strongly dependent on the phase difference *θ* of the roughness of the upper and lower plates. As the oscillation Reynolds number Re_Ω_ increases, the velocity profile and the average velocity *u_m_*(*t*) of AC EOF oscillate rapidly but the velocity amplitude decreases. The Deborah number *De* plays a similar role to Re_Ω_, which makes the AC EOF velocity profile more likely to oscillate. Meanwhile, phase lag *χ* (representing the phase difference between the electric field and the mean velocity) decreases when *G* and *θ* are increased. However, for larger *λ* (e.g., *λ* > 3), it almost has no phase lag *χ*.

## 1. Introduction

With the developments in microfluidic technology, the electroosmotic flow (EOF) [1] is used widely in chemical and biomedical fields, such as DNA separation, cell sorting, ion transport, sample separation, and mixing in microfluidic chips, etc. [2]. EOF is a specific phenomenon of electrodynamic flow in micro- or nano-scale channels. This flow phenomenon offers the advantages of requiring no external mechanical force, consuming low energy, and being simple to operate. Compared to the conventional-scale channels, the flow within micro-scale channels possesses its own special characteristics, such as relative surface roughness, micro-scale effects, slip effects, surface forces and capillary effects, rapid heat conduction effects, etc. [3]. Domestic and foreign scholars have conducted a large number of studies on the EOF of Newtonian [4,5,6,7,8,9,10] and non-Newtonian fluids [11,12,13,14] in smooth microchannels of different geometries with regard to theory, numerical simulation, and experimentation. In addition, the effect of Joule heating on the fluid is negligible in microchannels with a small cross-sectional area and high thermal conductivity [15].

The aforementioned studies all consider smooth wall EOF flows. Usually, the wall roughness of microchannels is induced by the manufacturing process of the device or the precipitation of other substances (such as macromolecules) on the wall. On the other hand, for all practical purposes, in order to boost the mixing efficiency of the fluid system at times, some roughness may be artificially designed on the channel walls. Therefore, the wall roughness in microchannels can be viewed as an intentionally designed feature rather than just an unavoidable defect. In microflow, the relative wall roughness (i.e., the ratio of wall roughness to channel radius) is increased due to the small size of a microchannel. Thus, the wall roughness can affect fluid flow in a microchannel. Different flow velocity modes will affect the component-separation efficiency, mixing reactions, flow rates, and heat-transfer process of the microfluidic systems. The effect of roughness on flow is a complex issue with pros and cons. At present, much of the research in this area by scholars is limited to the flow driven by electric field forces in smooth microchannels, and very few studies consider wall roughness. Since 1970, several scholars have considered the problem of laminar flow problems with rough walls. Wang [16] first researched the Stokes flow between flat plates with corrugated roughness. Chu [17] has applied the perturbation expansion method to study the effect of corrugated roughness on fluid flow. Malevich et al. [18] have investigated the theoretical knowledge of three-dimensional Couette flow between rough plates. Ng and Wang [19] obtained the exact solution of Darcy–Brinkman flow. Xia et al. [20] used the complex potential function and the boundary integral method to show the analytical solution of the EOF of a microchannel composed of parallel plates with one smooth wall and a sinusoidal ripple boundary on the other wall, and they analyzed the influence of ripple amplitude and the width between the two plates on the flow field. Shu et al. [21] utilized the boundary perturbation method (BPM) to tackle the EOF analytical solution of a parallel plate microchannel with longitudinal sinusoidal ripple boundaries, and they verified its accuracy. Cho et al. [22,23,24] employed the finite volume method (FVM) to study the DC/AC EOF of Newtonian fluid and power-law fluid between rough parallel plates with two superposed sinusoidal functions. In special cases, when degenerating into a single sinusoidal function to simulate ripples, the results obtained are consistent with those of Xia [20].

BPM has been widely used to study the EOF problem in microchannels with rough wall surfaces. For instance, Chang et al. [25] researched the EOF of circular microfluidic channels with axial roughness using BPM. The reason for the increase or decrease of the velocity is to take into account the influence of the relative roughness amplitude, wavenumber, and pressure gradient on the electric potential and velocity distribution. The increase in velocity is due to the fact that the EDL electric field force is larger than the electric field force at the center of the channel, and the pressure gradient also amplifies the flow velocity. Keramati et al. [26] have investigated the impact of corrugated roughness on fully developed EOF and heat transfer in circular microtubes, revealing that higher corrugation numbers and relative roughness result in reduced Nusselt numbers, indicating negative effects on both hydrodynamic and thermal features of EOF, while also identifying the Nusselt number correlation with decreasing joule heating rate and increasing dimensionless Debye–Hückel parameter. Messinger and Squires [27] found that when the wall conductivity is too high, the nano-scale wall roughness that usually appears on micromachined metal electrodes can significantly inhibit EOF. Zhang et al. [28] used molecular dynamics methods to numerically study the effect of wall roughness on micro-scale EOF. Fakhari and Mirbozorg [29] used FVM to numerically study the impact of sinusoidal, sawtooth, and square-tooth wall-surface roughness on EOF between parallel plates. It has been shown that the wall-surface roughness may degrade the velocity and thus reduce the EOF velocity. Buren et al. [30] have used BPM and studied electromagnetic hydrodynamic (EMHD) flow in micro-parallel channels with sinusoidal roughness under the Debye–Hückel approximation and have obtained perturbation solutions for velocity and electric potential. In addition, the EMHD of transverse and longitudinal roughness have been studied [31,32]. Recently, the effect of small-amplitude random lateral wall roughness on EMHD flow in microchannels in parallel plates [33] and cylindrical [34] microchannels was studied using the perturbation method of stationary random function theory. Hosham et al. [35] studied the heat and mass transfer effects in EOF based on the viscoplastic Bingham fluid model in complex corrugated microchannels. The flow and heat-transfer characteristics in a microchannel with a cross-section that periodically expands gradually and suddenly contracts was numerically studied by Zhu et al. [36]. In addition, microchannels with the same volume and a similar convective heat-transfer area are compared. The pressure and viscous resistance of the groove walls were analyzed analytically to explain why the groove can sustain a low microchannel pressure drop. Mohammadi et al. [37] have studied the effect of nanofluids as coolants and sinusoidal wave walls on the performance of rectangular microchannel heat sinks by using the FVF method, revealing that higher wave amplitudes and lower wavelengths improve heat-transfer performance.

In industrial and engineering applications, microfluidic devices are widely used to analyze biological fluids, which are frequently solutions of long-chain molecules that exhibit some non-Newtonian fluid properties. Therefore, it is of practical interest to study non-Newtonian fluids in microchannels. Martínez et al. [38] simplified the PTT fluid control equation through lubrication theory and then used BPM to resolve the simplified PTT fluid EOF approximate analytical solution in a parallel plate with transverse sinusoidal roughness, but the result only retained first-order accuracy. Si and Jian [39] utilized the BPM to provide approximate analytical solutions for the velocity and volumetric flow rates of periodic EMHD flows involving a conductive, incompressible, and viscous Jeffrey fluid within micro-parallel plates featuring sinusoidal roughness.

In summary, there has not been enough attention paid by domestic and international scholars to the EOF of non-Newtonian fluids with roughness in microchannels. In particular, studies using theoretical analysis and numerical simulations are still in the development stage, and there are a large number of fundamental and mechanistic systematic studies that need to be carried out. Based on this status quo, in this paper, we studied the effect of periodic EOF of Maxwell fluid in parallel plate microchannels with sinusoidal roughness using the linearized Poisson–Boltzmann equation and the Cauchy momentum equation. The paper is organized as follows: Section 2 presents the mathematical modeling, results and comparisons are discussed in Section 3; and the conclusions are drawn in Section 4.

## 2. Mathematical Modeling

### 2.1. Description of the Problem

**Figure 1:**
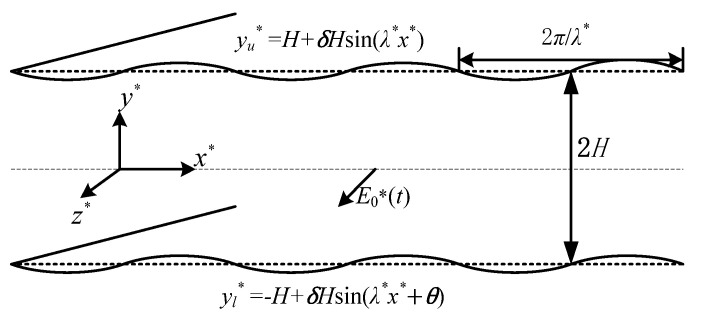
Schematic of AC EOF through a microchannel with sinusoidal wavy walls.

The mathematical model of the AC EOF for an incompressible, linear, and viscoelastic Maxwell fluid in a microchannel is considered in Figure 1. The mean height of the microchannel is 2*H*. The length and width are much larger than the height of the microchannel. As shown in the rectangular coordinate system (x*, y*, z*), the x* axis is located in the middle of the plate, and an AC electric field with a strength of E*_0_ and periodic pressure are applied at both ends of the channel. At this time, the fluid is considered to be along the z* direction of flow. The applied electric field strength is significantly lower than 10^5^ V/m. Additionally, the flow system is not expected to reach a chaotic state due to the time scale associated with electromigration in the EDL, which is estimated to be within the range of 10^−8^~10^−7^ s [12]. This time scale is at least two orders of magnitude smaller than the EOF, ranging from 10^−5^~10^−3^ s. Therefore, the transient effects of the EDL can be disregarded. Due to the small channel cross-sectional area and high thermal conductivity of silica, we assume negligible Joule heating effects and assume that fluid properties are independent of temperature variations. The corrugated wall surface of the lower plate and the upper plate are, respectively, expressed as [30]

(1) $${y_{l}}^{*}=H\left[-1+\delta \sin\left(\lambda^{*}x^{*}+\theta \right)\right],\;y_{u}^{*}=H\left[1+\delta \sin\left(\lambda^{*}x^{*}\right)\right],$$

where, *δ* is the ratio of the ripple amplitude to the average half-height of the channel, λ* is the wave number, and *θ* is the phase difference.

### 2.2. Mathematical Models and Approximate Solutions

According to the electric double layer theory, the relationship between the electric potential Ψ(x*, y*) and the net charge density ρ*_e_(x*, y*) can be described by the Poisson–Boltzmann (P-B) equation [1]

(2) $$\nabla^{*2}\Psi =-\frac{\rho_{e}^{*}}{\varepsilon },$$

(3) $$\rho_{e}^{*}=-2n_{0}z_{v}e\sin h\frac{z_{v}e\Psi }{k_{b}T},$$

where, *z_ν_*, *e*, *n*_0_, *ε*, *k_b_*, and *T*, respectively, represent the valence, the charge carried by the electron, the concentration of liquid ions, the dielectric constant of the electrolyte solution, the Boltzmann constant, and the absolute temperature.

If Ψ is small enough (<<25 mV), namely *z_v_e*Ψ/(*k_b_T*) << 1, the term sinh(*z_v_e*Ψ/(*k_b_T*)) ≈ *z_v_e*Ψ/(*k_b_T*). This linearization is called Debye–Hückel linearization (Physically, it means that the electric potential energy is very small compared with the thermal energy of ions). According to Equations (2) and (3), the linearized P-B equation is obtained

(4) $$\frac{\partial^{2}\Psi }{\partial x^{*2}}+\frac{\partial^{2}\Psi }{\partial y^{*2}}=\kappa^{2}\Psi .$$

The corresponding boundary conditions are given as

$$\Psi (x^{*},y^{*})=\zeta_{u},\;\mathrm{at}\;y={y_{u}}^{*}$$

(5) $$\Psi (x^{*},y^{*})=\zeta_{l},\;\mathrm{at}\;y={y_{l}}^{*}$$

here we assume that *ζ_u_* and *ζ_l_* are constants [21].

An incompressible Maxwell fluid should satisfy the continuity and Cauchy momentum equations

(6) $$\nabla^{*}\cdot U=0,$$

(7) $$\rho \left(\frac{\partial U}{\partial t^{*}}+\left(U\cdot \nabla^{*}\right)U\right)=-\nabla^{*}P+\nabla^{*}\cdot \tau +\rho_{e}^{*}E_{0}^{*}(t)e_{z^{*}}.$$

The generalized Maxwell fluid constitutive relationship of linear viscoelasticity is as follows [12,40]

(8) $$\tau +\lambda_{1}\frac{\partial \tau }{\partial t^{*}}=\eta_{0}[\nabla^{*}U+{\left(\nabla^{*}U\right)}^{T}].$$

It is considered that there is only flow along the z* direction (flow velocity **U** = (0, 0, *W*)). According to the incompressibility condition (6), the convection term in the Cauchy momentum Equation (7) will disappear. Combined with the constitutive Equation (8), the operator 1 + *λ*_1_*∂*/(∂t*) acts on both sides of the Cauchy momentum Equation (7), and the governing equation is simplified as follows

(9) $$\rho \left(1+\lambda_{1}\frac{\partial }{\partial t^{*}}\right)\frac{\partial W}{\partial t^{*}}=-\left(1+\lambda_{1}\frac{\partial }{\partial t^{*}}\right)\frac{\partial P}{\partial z^{*}}+\eta_{0}\nabla^{*2}W+\rho_{e}^{*}(x,y)\left(1+\lambda_{1}\frac{\partial }{\partial t^{*}}\right)E_{0}^{*}(t).$$

Assume that the velocity and pressure of the alternating electric field and the periodic EOF can be written in the following complex functional form

(10) $$E_{0}^{*}\left(t^{*}\right)=ℜ\left\{E_{0}e^{i\omega t^{*}}\right\},\;W\left(x^{*},y^{*},t^{*}\right)=ℜ\left\{w^{*}\left(x^{*},y^{*}\right)e^{i\omega t^{*}}\right\},\;P\left(z^{*},t^{*}\right)=ℜ\left\{p^{*}\left(z^{*}\right)e^{i\omega t^{*}}\right\},$$

then, Equation (9) can be simplified to

(11) $$i\omega \rho \left(1+i\lambda_{1}\omega \right)w^{*}=-\left(1+i\lambda_{1}\omega \right)\frac{\partial p^{*}}{\partial z^{*}}+\eta_{0}\nabla^{*2}w^{*}+\left(1+i\lambda_{1}\omega \right)\rho_{e}^{*}E_{0}.$$

The boundary conditions of the upper and lower walls of the channel corresponding to Equation (11) are

(12) $$w^{*}(x^{*},y^{*})=0,\;\mathrm{at}\;y={y_{u}}^{*},\;y={y_{l}}^{*}$$

Introduce a set of dimensionless parameters:

(13) y=y∗H, K=κH, ζ=ζlζu, φ=Ψζu, w=w∗Ueo, Ueo=−εζuE0η0, ReΩ=ρωH2η0, G=−H2η0Ueo∂p∗∂z∗, De=λ1ω,

In the above formula, the dimensionless electric width *K* represents the ratio of the half-height (*H*) of the microchannel to the Debye length (1/*κ*, where *κ* = *z_v_e*(2*n*_0_/*εk_b_T*)^1/2^); the physical meaning of the oscillation Reynolds number Re_Ω_ is the ratio of the diffusion time scale (*t_diff_* = *ρH*^2^/*η*_0_) and the period of the external electric field (*t_E_* = 1/*ω*); Deborah number *De* represents the ratio of the relaxation time *λ*_1_ of the fluid to the vibration time 1/*ω* of the electric field; and *G* represents the dimensionless pressure gradient applied in the axial direction of the channel.

Substituting the dimensionless parameter (13) into the P-B Equation (2), the EOF control Equation (10) and the boundary conditions (3) and (12), the equation satisfied by corresponding dimensionless electrical potential *φ*(*x*, *y*) and velocity *w*(*x*, *y*) is

(14) $$\frac{\partial^{2}\varphi }{\partial x^{2}}+\frac{\partial^{2}\varphi }{\partial y^{2}}=K^{2}\varphi ,$$

(15) $$\frac{\partial^{2}w}{\partial x^{2}}+\frac{\partial^{2}w}{\partial y^{2}}-{\mathrm{iRe}}_{\Omega }(1+iDe)w=-(1+iDe)G-K^{2}(1+iDe)\varphi .$$

The corresponding boundary conditions are

$$\varphi \left(x,y\right)=1,\;w\left(x,y\right)=0,\;\mathrm{at}\;y=y_{u},$$

(16) $$\;\varphi \left(x,y\right)=\zeta ,\;w\left(x,y\right)=0,\;\mathrm{at}\;y=y_{l}.$$

In the absence of roughness, the electrical potential *φ* and velocity *w* are only functions of *y*. However, the presence of wall roughness causes a function change in the *x* direction. In the following analysis, it is assumed that *δ* << 1 and the electrical potential *φ* and velocity *w* expanded to the power of *δ* as

(17) $$R\left(x,y\right)=R_{0}\left(y\right)+\delta R_{1}\left(x,y\right)+\delta^{2}R_{2}\left(x,y\right)+\cdots ,$$

The Taylor expansion of the function *R*, at *y* = 1 and *y* = −1 on the upper wall *y* = *y_u_* and the lower wall *y* = *y_l_*, respectively, is

$$R\left(x,1+\delta \sin(\lambda x)\right)=R\left(x,1\right)+\delta \sin(\lambda x)R_{y}\left(x,1\right)+\frac{\delta^{2}\sin^{2}(\lambda x)}{2}R_{yy}\left(x,1\right)+\cdots$$

$$=R_{0}\left(1\right)+\delta [\sin\left(\lambda \theta \right){R^{\prime }}_{0}\left(1\right)+R_{1}\left(x,1\right)]+\delta^{2}[\frac{\sin^{2}\left(\lambda \theta \right)}{2}{R^{\prime \prime }}_{0}(1)+\sin\left(\lambda \theta \right)R_{1y}\left(x,1\right)+R_{2}\left(x,1\right)]+O(\delta^{3}).$$

$$R\left(x,-1+\delta \sin(\lambda x+\theta )\right)=R\left(x,-1\right)+\delta \sin(\lambda x+\theta )R_{y}\left(x,-1\right)+\frac{\delta^{2}\sin^{2}(\lambda x+\theta )}{2}R_{yy}\left(x,-1\right)+\cdots$$

(18) $$=R_{0}\left(-1\right)+\delta [\sin\left(\lambda x+\theta \right){R^{\prime }}_{0}\left(-1\right)+R_{1}\left(x,-1\right)]+\delta^{2}[\frac{\sin^{2}\left(\lambda x+\theta \right)}{2}{R^{\prime \prime }}_{0}\left(-1\right)+\sin\left(\lambda x+\theta \right)R_{1y}\left(x,-1\right)+R_{2}\left(x,-1\right)]+\cdots .$$

Substituting Equation (17) into Equations (14) and (15), we obtain the boundary value problem of the differential equation that is satisfied by the power of *δ*

(19) δ0: d2φ0dy2=K2φ0, d2w0dy2−iReΩ(1+iDe)w0=−(1+iDe)G−K2(1+iDe)φ0.

(20) δ1: ∂2φ1∂x2+∂2φ1∂y2=K2φ1, ∂2w1∂x2+∂2w1∂y2−iReΩ(1+iDe)w1=−K2(1+iDe)φ1.

(21) δ2: ∂2φ2∂x2+∂2φ2∂y2=K2φ2, ∂2w2∂x2+∂2w2∂y2−iReΩ(1+iDe)w2=−K2(1+iDe)φ2.

The corresponding boundary condition (16) uses the Taylor expansion of the function *R* at *y* = ±1 (18), and it can be obtained that the following boundary conditions are

(22) $$\delta^{0}:\varphi_{0}\left(1\right)=1,\;\varphi_{0}\left(-1\right)=\zeta ,\;w_{0}\left(1\right)=0,\;w_{0}\left(-1\right)=0,$$

(23) $$\delta^{1}:\varphi_{1}\left(x,1\right)=-\sin(\lambda x){\varphi^{\prime }}_{0}\left(1\right),\;\varphi_{1}\left(x,-1\right)=-\sin(\lambda x+\theta ){\varphi^{\prime }}_{0}\left(-1\right),\;w_{1}\left(x,1\right)=-\sin(\lambda x){w^{\prime }}_{0}\left(1\right),\;w_{1}\left(x,-1\right)=-\sin(\lambda x+\theta ){w^{\prime }}_{0}\left(-1\right),$$

$$\;\delta^{2}:\;\varphi_{2}\left(x,1\right)=-\frac{\sin^{2}\left(\lambda x\right)}{2}{\varphi^{\prime \prime }}_{0}\left(1\right)-\sin\left(\lambda x\right)\frac{\partial \varphi_{1}}{\partial y}{|}_{y=1},\;\varphi_{2}\left(x,-1\right)=-\frac{\sin^{2}\left(\lambda x+\theta \right)}{2}{\varphi^{\prime \prime }}_{0}\left(-1\right)-\sin(\lambda x+\theta )\frac{\partial \varphi_{1}}{\partial y}{|}_{y=-1},$$

(24) $$\;w_{2}\left(x,1\right)=-\frac{\sin^{2}\left(\lambda x\right)}{2}{w^{\prime \prime }}_{0}\left(1\right)-\sin\left(\lambda x\right)\frac{\partial w_{1}}{\partial y}{|}_{y=1},w_{2}\left(x,-1\right)=-\frac{\sin^{2}\left(\lambda x+\theta \right)}{2}{w^{\prime \prime }}_{0}\left(-1\right)-\sin\left(\lambda x+\theta \right)\frac{\partial w_{1}}{\partial y}{|}_{y=-1}.$$

where

(25) iReΩ1+iDe=α0+iβ02,

then it can be calculated

(26) α0=(ReΩ2)1/2[1+De2−De]1/2, β0=(ReΩ2)1/2[1+De2+De]1/2,

combining Equations (19) and (22), we can calculate

$$\varphi_{0}(y)=A_{0}\cosh\left(Ky\right)+B_{0}\sinh(Ky),$$

(27) w0y=C0coshα0+iβ0y+D0sinhα0+iβ0y+a0coshKy+b0sinhKy+GiReΩ,

Substituting Equation (22) into (27), the undetermined constants *A*_0_, *B*_0_, *C*_0_, *D*_0_, *a*_0_, *b*_0_ can be obtained. See Appendix A.

According to the boundary condition (23), the solution expression of Equation (20) can be written as

(28) $$\varphi_{1}\left(x,y\right)=\;f_{1}(y)\sin\left(\lambda x\right)+g_{1}(y)\cos(\lambda x),\;w_{1}\left(x,y\right)=\;F_{1}(y)\sin\left(\lambda x\right)+G_{1}(y)\cos(\lambda x).$$

Substituting Equation (28) into Equation (20), we can obtain

$$f_{1}\left(y\right)=A_{1}\cosh(K_{1}y)+B_{1}\sinh(K_{1}y),\;\;g_{1}\left(r\right)=A_{2}\cosh(K_{1}y)+B_{2}\sinh(K_{1}y),$$

$$F_{1}\left(y\right)=C_{1}\cosh\left(\alpha_{1}+i\beta_{1}\right)y+D_{1}\sinh\left(\alpha_{1}+i\beta_{1}\right)y+a_{1}\cosh(K_{1}y)+b_{1}\sinh(K_{1}y),$$

(29) $$G_{1}\left(y\right)=C_{2}\cosh\left(\alpha_{1}+i\beta_{1}\right)y+D_{2}\sinh\left(\alpha_{1}+i\beta_{1}\right)y+a_{2}\cosh(K_{1}y)+b_{2}\sinh(K_{1}y).$$

where $\;K_{1}^{2}=K^{2}+\lambda^{2},\;{\left(\alpha_{1}+i\beta_{1}\right)}^{2}={\left(\alpha_{0}+i\beta_{0}\right)}^{2}+\lambda^{2}$ , the undetermined constants *A_j_*, *B_j_*, *C_j_*, *D_j_*, *a_j_*, *b_j_* (*j* = 1, 2), see Appendix A.

According to the boundary condition (24), the solution expression of Equation (21) can be given as follows

$$\varphi_{2}\left(x,y\right)=\;h\left(y\right)+f_{2}(y)\sin\left(2\lambda x\right)+g_{2}(y)\cos(2\lambda x),$$

(30) $$w_{2}\left(x,y\right)=H\left(y\right)+\;F_{2}(y)\sin\left(2\lambda x\right)+G_{2}(y)\cos(2\lambda x).$$

Substituting Equation (30) into Equation (21), we can obtain

$$h\left(y\right)=A_{3}\cosh\left(Ky\right)+B_{3}\sinh(Ky),\;f_{2}\left(y\right)=A_{4}\cosh\left(K_{2}y\right)+B_{4}\sinh(K_{2}y),$$

$$g_{2}(y)=A_{5}\cosh(K_{2}y)+B_{5}\sinh(K_{2}y),$$

$$H\left(y\right)=C_{3}\cosh\left[\left(\alpha_{0}+i\beta_{0}\right)y\right]+D_{3}\sinh\left[\left(\alpha_{0}+i\beta_{0}\right)y\right]+a_{3}\cosh\left(Ky\right)+b_{3}\sinh(Ky),$$

$$F_{2}\left(y\right)=C_{4}\cosh\left[\left(\alpha_{2}+i\beta_{2}\right)y\right]+D_{4}\sinh\left[\left(\alpha_{2}+i\beta_{2}\right)y\right]+a_{4}\cosh\left(K_{2}y\right)+b_{4}\sinh(K_{2}y),$$

(31) $$G_{2}\left(y\right)=C_{5}\cosh\left[\left(\alpha_{2}+i\beta_{2}\right)y\right]+D_{5}\sinh\left[\left(\alpha_{2}+i\beta_{2}\right)y\right]+a_{5}\cosh\left(K_{2}y\right)+b_{5}\sinh(K_{2}y).$$

where $\;K_{2}^{2}=K^{2}+4\lambda^{2},\;{\left(\alpha_{2}+i\beta_{2}\right)}^{2}={\left(\alpha_{0}+i\beta_{0}\right)}^{2}+4\lambda^{2},$ the undetermined constants *A_j_*, *B_j_*, *C_j_*, *D_j_*, *a_j_*, *b_j_* (*j* = 3, 4, 5), see Appendix A.

### 2.3. Average Velocity

The flow velocity per unit width through the microchannel is averaged over one wavelength of wall roughness, and the complex amplitude of the average velocity can be derived

(32) $$\bar{u}=\frac{\lambda }{4\pi }\int_{0}^{\frac{2\pi }{\lambda }}dx\int_{-1+\delta \sin(\lambda x+\theta )}^{1+\delta \sin(\lambda x)}w\left(x,y\right)dy=\frac{\lambda }{4\pi }\int_{0}^{\frac{2\pi }{\lambda }}\{\int_{-1}^{1}w\left(x,y\right)dy+\int_{1}^{1+\delta \sin(\lambda x)}w\left(x,y\right)dy+\int_{-1+\delta \sin(\lambda x+\theta )}^{-1}w\left(x,y\right)dy\}dx=u_{0m}+\delta^{2}u_{2m}+o(\delta^{2})$$

where u0m=C0α0+iβ0sinhα0+iβ0+a0KsinhK+GiReΩ,

(33) $$u_{2m}=\frac{C_{3}}{\left(\alpha_{0}+i\beta_{0}\right)}\sinh\left(\alpha_{0}+i\beta_{0}\right)+\frac{a_{3}}{K}\sinh K+\frac{1}{4}\left[F_{1}\left(1\right)+{w^{\prime }}_{0}\left(1\right)-F_{1}\left(-1\right)\cos\theta -G_{1}\left(-1\right)\sin\theta -{w^{\prime }}_{0}\left(-1\right)\right].$$

Note that the Taylor expansion at *y* = ±1 was used in the last two integral calculations of Equation (32).

Therefore, the average velocity of AC EOF in the rough microchannel can be expressed as

(34) $$u_{m}\left(t\right)=ℜ\left\{\bar{u}\exp\left(it\right)\right\}=\left|U_{m}\right|\cos(t+\chi ).$$

where *χ* is the main argument of the average velocity $\bar{u}$ **,** which also represents the phase difference between the electric field and the average velocity, which is called phase lag.

## 3. Results and Discussion

In this section, we resolved the approximation of AC EOF for Maxwell fluid in a microchannel with sinusoidal roughness. The findings are mainly influenced by the following dimensionless parameters: electrodynamic width *K* (parameters related to the thickness of the electric double layer), the ratio of the ripple amplitude to the average half-height of the channel, the oscillation Reynolds number Re_Ω_, the zeta potential ratio *ζ* between the upper and lower walls, the wave number *λ* of the rough wall, the phase difference *θ* between the rough walls, the dimensionless pressure gradient *G*, and the Deborah number *De*. In engineering, in order to tackle practical problems, dimensionless parameters need to be converted into dimensioned parameters. The relaxation time must be smaller than the oscillation period (observation time), that is, it must satisfy *λ*_1_ < 2π/*ω* or *De* < 2π. Typically, the value of EDL thickness 1/*κ* at room temperature is 10^−7^ m to 5 × 10^−7^ m. Moreover, the linearized P-B equation is valid when the wall zeta potential is less than 25 mV. Therefore, the range of the Helmholtz–Smoluchowski EOF velocity Ueo of a Newtonian fluid is about 10^−5^ to 2.5 × 10^−4^ ms^−1^. From the condition *λ*_1_*U_eo_*/(1/*κ*) << 1, we determined that the effective range of relaxation time *λ*_1_ is 4 × 10^−4^ s to 5 × 10^−2^ s. Some typical parameter value ranges are as follows [10,11,12,13,14]: *H* = 100 µm, *ρ* = 10^3^ kg·m^−3^, *η*_0_ = 10^−3^ kgm^−1^s^−1^. At the same time, the applied electric field frequency ranges from 0 to 1.6 kHz, and the variation range of the corresponding angular frequency *ω* is from 0 to 10^4^ s^−1^. Therefore, the oscillation Reynolds number Re_Ω_ could take a value between 0 and 100. According to references [12,39], the value range of the relaxation time *λ*_1_ is set from 10^−4^ s to 10^3^ s.

**Figure 2:**
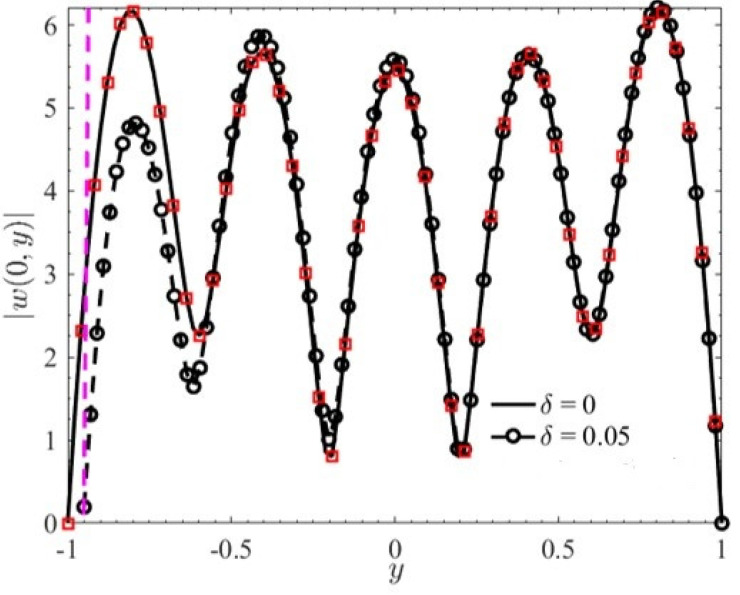
The amplitude of velocity |*w*(*x*, *y*)| with *y* (*K* = 10, *λ* = 8, *G* = 0, Re_Ω_ = 10, *θ* = 0.5*π*) (red square—Liu et al. [12]).

Figure 2 plots the AC EOF velocity amplitude for a Maxwell fluid for a given *x* = 0. As can be seen from Figure 2, the velocity amplitude is smaller in the rough microchannel than that in the smooth microchannel. The reason for this is that the increased contact area between the corrugation on the lower wall and the fluid causes the flow resistance of the fluid to increase, which reduces the amplitude of the velocity. In addition, the velocity profile in the smooth microchannel shows central symmetry, which is consistent with the results obtained by Liu et al. [12].

**Figure 3:**
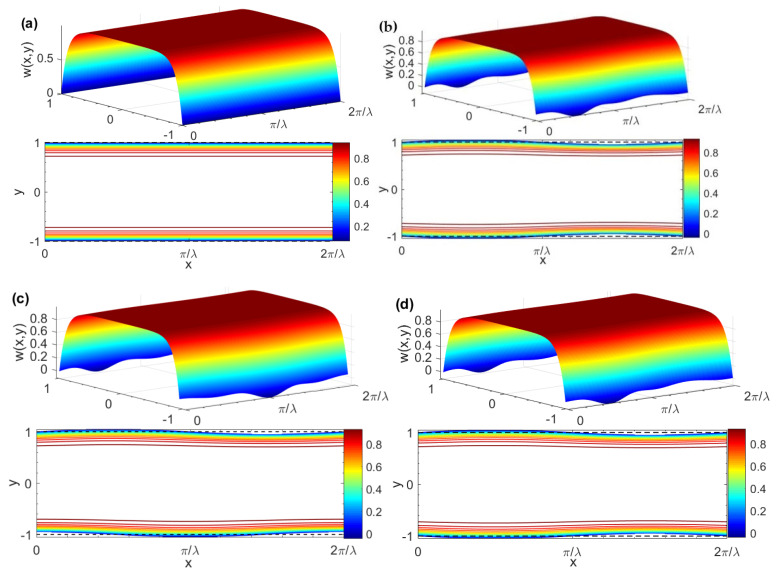
Three-dimensional AC EOF velocity distributions and contours for different *δ* and *θ* (*G* = 0, *K* = 10, *λ* = 8, *ζ* = 1, *δ* = 0.05, *De* = 0, Re_Ω_→0). (**a**) AC EOF velocity distribution in the smooth microchannel; (**b**) *θ* = 0; (**c**) *θ* = *π*/2; (**d**) *θ* = *π*.

Figure 3 depicts the three-dimensional pure AC EOF velocity and contour distribution diagram of a Newtonian fluid corresponding to different δ and θ. Figure 3a shows the velocity diagram under smooth channel (i.e., *δ* = 0). It can be seen from Figure 3 that, especially near the wall, as the dimensionless ripple amplitude *δ* increases the fluctuations in the Newtonian fluid velocity profile become more and more obvious, and the velocity distribution depends on the phase difference *θ* of the upper and lower plate roughness ripples. In addition, it can also be seen that the classic pin-shaped velocity profile similar to this is also presented.

**Figure 4:**
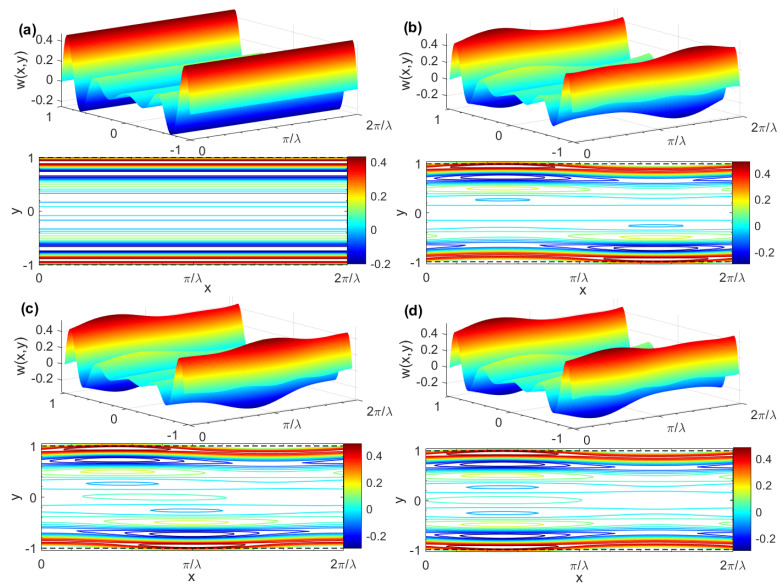
Three-dimensional AC EOF velocity distributions and contours for different *δ* and *θ* (*G* = 0, *K* = 10, *λ* = 8, *ζ* = 1, *De* = 2, Re_Ω_ = 100). (**a**) *δ* = 0, *θ* = 0; (**b**) *δ* = 0.05, *θ* = 0; (**c**) *δ* = 0.05, *θ* = *π*/2; (**d**) *δ* = 0.05, *θ* = *π*.

**Figure 5:**
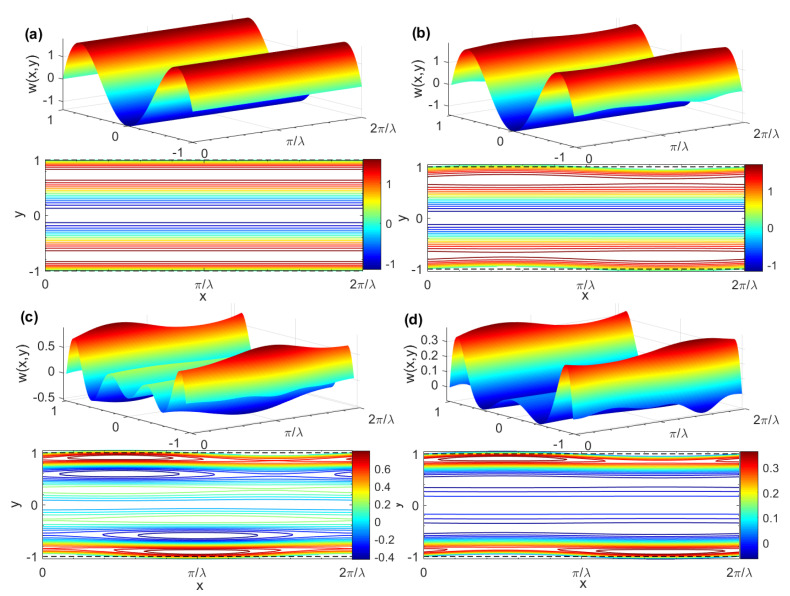
Three-dimensional AC EOF velocity distributions and contours for different *δ*, *De* and Re_Ω_ (*G* = 0, *K* = 10, *λ* = 8, *ζ* = 1, *θ* = 0). (**a**) *δ* = 0, *De* = 2, Re_Ω_ = 10; (**b**) *δ* = 0.05, *De* = 2, Re_Ω_ = 10; (**c**) *δ* = 0.05, *De* = 2, Re_Ω_ = 50; (**d**) *δ* = 0.05, De = 0.5, Re_Ω_ = 50.

Figure 4 displays the three-dimensional pure AC EOF velocity and contour distribution map of a Maxwell fluid corresponding to different *δ* and *θ*. The velocity distribution under a smooth channel (i.e., *δ* = 0) is shown in Figure 4a. For a given *δ* = 0.05, it is easy to see from Figure 4b,d that when the rough wall changes from in-phase (*θ* = 0) to out-of-phase (*θ* = *π*), the velocity profile is significantly affected by the wall roughness and obvious wave phenomena appeared. In addition, the velocity distribution depends on the phase difference *θ* of the upper and lower plates’ roughness ripples. Combined with Figure 5, it is easy to see that for a given *G*, *K*, *λ*, *ζ*, *De*, and *θ*, the velocity profile oscillates rapidly, and the amplitude decreases with the increase of Re_Ω_. Moreover, the amplitude is larger in the narrow EDL region near the wall and gradually decreases away from the EDL region. The reason for this is that the vibrational period is much shorter than the dissipation timescale and the fluid motion does not have enough time to diffuse to the plane in the middle of the two walls of the microchannel. In addition, for linear generalized Maxwell fluids, the larger the *De*, the smaller the resummation capacity and the larger the elastic effect. The velocity profile is more likely to oscillate under the action of an external electric field, which is caused by Maxwell’s “fading memory” phenomenon.

**Figure 6:**
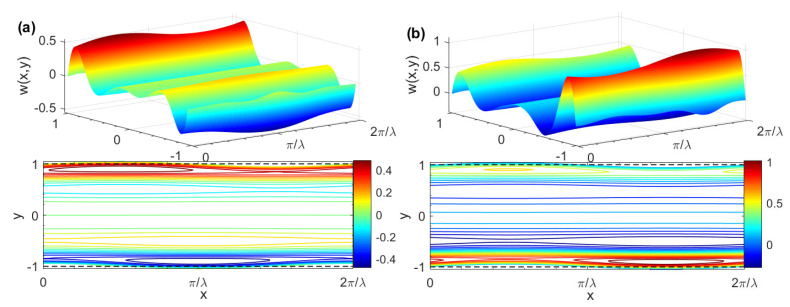
Three-dimensional AC EOF velocity distributions and contours for different *ζ* (*G* = 0, *K* = 10, *λ* = 8, *De* = 1, Re_Ω_ = 50, *θ* = 0, *δ* = 0.05). (**a**) *ζ* = −1; (**b**) *ζ* = 2.

Figure 6 reveals the three-dimensional pure AC EOF velocity and contour distribution diagram of a Maxwell fluid corresponding to different *δ*. When the two parallel plate walls possess opposite charges (i.e., *ζ* = −1), the direction of the EOF in the micro-parallel channel is directly related to the polarity of the charge on the channel wall. This conclusion is similar to the outcome obtained in reference [8]. It is easy to see from Figure 6 that when the *ζ* potential of the lower plate is high (i.e., *ζ* = 2), the velocity will rapidly increase from zero to the maximum value in the EDL region of the lower wall. Subsequently, the velocity will decrease with the distance away from the lower plate wall, gradually increasing in the EDL region of the upper wall as the velocity reaches zero on the upper wall surface, which can be attributed to the fact that the flow of the fluid is driven by the electroosmotic force result from the interaction of the external electric field and the EDL. This due to the higher ion concentration in the EDL, which causes the fluid to flow more violently in the EDL region of the lower parallel plate wall. In the EDL region of the upper parallel plate wall, the ion concentration is low and the fluid flow is relatively slow.

**Figure 7:**
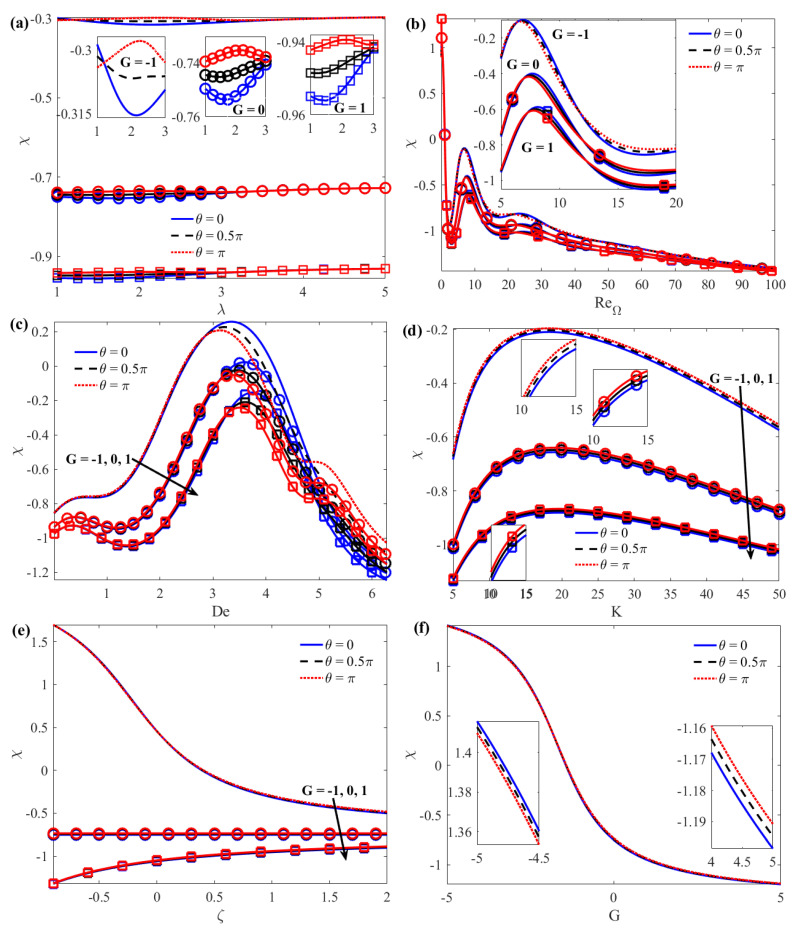
Variations of the phase lag *χ* between the mean velocity and electric field potential with (**a**) *λ*; (**b**) Re_Ω_; (**c**) *De*; (**d**) *K*; (**e**) *ζ*; and (**f**) *G* when *K* = 10, *ζ* = 1, *De* = 2, Re_Ω_ = 5, *λ* = 2, and *δ* = 0.1.

From Equation (32), it can be seen that *u*_2*m*_ > 0 or *u*_2*m*_ < 0 correspond to an increase or decrease, respectively, of the mean velocity in the microchannels with roughness compared to that in the smooth channels. In AC EOF, there is a time difference *χ* (called phase lag) between the velocity and the applied electric field, which represents the time required for momentum diffusion. Figure 7 indicates the variation of phase lag *χ* with *λ*, Re_Ω_, *De*, *K*, and *ζ* at different *G* and *θ*, and the changes of *χ* with *G* at different *θ*. As expected, the phase lag *χ* reduces with augmented *G*. The reason for this is that the forward pressure gradient promotes flow while the reverse pressure gradient hinders flow. For larger wave numbers (such as *λ* > 3), there is almost no phase lag *χ* between the electric field and the average velocity, which increases with the increase of *θ* and is more obvious for smaller wave numbers (such as *λ* ≤ 3). This conclusion is consistent with the results of the literature [28] (Figure 7a). For a given Re_Ω_, the phase lag *χ* also increases or decreases significantly with *θ*. On the other hand, the results show that the velocity profile oscillates rapidly with the increase of Re_Ω_ and the amplitude becomes smaller. Under certain parameters, the same Re_Ω_ number will produce the same *χ* peak value (as shown in Figure 7b). The reason for this is that the rough wall changes from in-phase (*θ* = 0) to the out-of-phase (*θ* = *π*). Although the increased surface area of the flowing fluid in contact with the solid wall results in greater resistance, the shear thinning effect of a Maxwell fluid exhibits faster flow (as shown in Figure 7c). The phase lag *χ* first increases and then decreases with the increase of *K*. The increase of *K* means that the thickness of the EDL decreases. When the EDL is very thin, ions only need to overcome a small potential difference to reach the electrode surface. At this point, the flow velocity is faster, but the motion of the fluid on the microchannel walls is affected by ripples and roughness, resulting in a larger flow resistance. When the EDL is thick (small *K*), the effect is opposite. Therefore, in rough corrugated microchannels, the appropriate EDL thickness is selected according to specific conditions to achieve the required fluid control and transmission effects (Figure 7d). The phase lag *χ* varies with *ζ* and *G* and there is almost no phase lag (Figure 7e,f).

**Figure 8:**
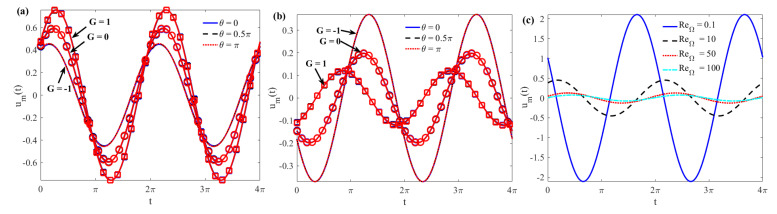
Variations of *u_m_*(*t*) with *t* (**a**) *ζ* = 2, *De* = 2, Re_Ω_ = 5; (**b**) *ζ* = −2, *De* = 2, Re_Ω_ = 5; and (**c**) *θ* = 0, *G* = 0, *ζ* = 1 when *K* = 10, *λ* = 2, and *δ* = 0.1.

Figure 8a,b expresses the average velocity *u_m_*(*t*) of AC EOF in different *θ* and *G* for rough microchannels as a function of *t*. For a given *ζ*, it is easy to see in the figure that the velocity profile becomes larger when the pressure gradient changes from a reverse pressure gradient (*G* = −1) to a forward pressure gradient (*G* = 1). This demonstrates that the pressure gradient hinders or promotes the flow (see Figure 8a). When the two parallel plate walls are oppositely charged, the electroosmotic force becomes resistance, so the velocity profile along the pressure gradient near the lower plate is smaller than the velocity profile reverse pressure gradient (see Figure 8b). It can also be seen from the figure that there is no phase lag phenomenon in different *θ* velocity profiles. For given parameters, the average velocity *u_m_*(*t*) oscillates rapidly with the increase of Re_Ω_ and the amplitude becomes smaller (see Figure 8c), which is consistent with the previous known results (see Figure 4). It can also be seen from Figure 8 that the average velocity *u_m_*(*t*) oscillates periodically with *t*, that the period is consistent with the external electric field, and that no phase lag appears.

**Figure 9:**
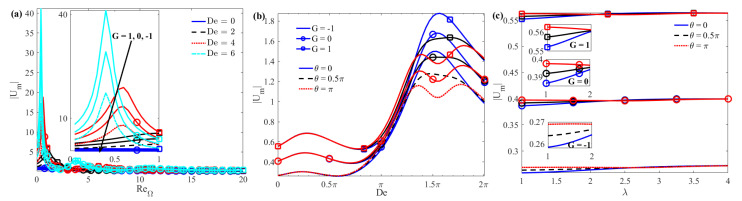
The complex amplitude of the average velocity |*U_m_*| as a function of (**a**) Re_Ω_; (**b**) *De*; and (**c**) *λ* when *K* = 10, *ζ* = 1, and *δ* = 0.1.

The amplitude of the mean velocity |*U_m_*| as a function of the parameters Re_Ω_, *De*, and *λ* is given in Figure 9. For a given *K*, a larger Re_Ω_ and a smaller *G* will result in a smaller |*U_m_*| (see Figure 9a). For a given Re_Ω_, the same *De* will result in the same peak value of |*U_m_*|. This is because the elastic effect of the fluid will cause the AC EOF velocity to increase for some *De* numbers and decrease for other*s*. In addition, under a larger elastic effect (that is, a larger *De* number), |*U_m_*| decreases with the increase of the wall roughness phase difference *θ* between the upper and lower plates. The reason for this is that when the rough wall changes from in-phase (*θ* = 0) to out-of-phase (*θ* = *π*), the area of the wall where the flowing fluid contacts the solid increases, resulting in greater resistance (Figure 9b). |*U_m_*| does not change significantly with increasing wave number. For larger wave numbers (such as *λ* ≥ 2.5), the impact of the roughness phase difference *θ* on |*U_m_*| is negligible (Figure 9c).

## 4. Conclusions

In this section, the AC EOF problem for a Maxwell fluid between micro-parallel plates with sinusoidal wall roughness is studied using the boundary perturbation expansion method and the linear superposition principle. From the above theoretical and drawing analysis, the following conclusions can be drawn:

The velocity distribution of the fluid is significantly affected by the wall roughness, there is an obvious fluctuation phenomenon, and the velocity distribution depends on *θ*. The velocity profile oscillates rapidly with the increase of Re_Ω_ and the amplitude becomes smaller. The amplitude is larger in the narrow EDL region near the wall and gradually decreases away from the EDL region. For some given parameters, the larger the *De* is, the easier it is for the velocity profile to oscillate under the action of an external electric field. When the *ζ* potential is high, the velocity rapidly increases from zero to a maximum value in the EDL region. The phase lag *χ* diminishes with the growth of *G*. For larger wave numbers (such as *λ* > 3), there is almost no *χ* between the electric field and the average velocity. For a given Re_Ω_, the *χ* also increases or decreases significantly with *θ*. On the other hand, it shows that the velocity profile oscillates rapidly and the amplitude becomes smaller and smaller as Re_Ω_ increases. For a given larger *De* number, a different *θ* has a more obvious *χ*, and *χ* declines as *θ* improves. *χ* first increases and then lessens with the enhancement of *K*, and there is almost no phase lag phenomenon with *ζ* and *G*. The velocity profile of the average velocity *u_m_*(*t*) increases with the increase of *G*, and there is no phase lag in different *θ* velocity profiles. The *u_m_*(*t*) oscillates rapidly and the amplitude decreases with the increase of Re_Ω_. For a given *K*, a larger Re_Ω_ and a smaller *G* result in a smaller average velocity amplitude |*U_m_*|. For a given Re_Ω_, the same number of *De* will produce the same |*U_m_*| peak value. For larger *De* numbers, |*U_m_*| reduces as *θ* enhances. |*U_m_*| has no obvious change with the augmentation of wave number *λ*. For larger wave numbers (such as *λ* ≥ 2.5), the influence of *θ* on |*U_m_*| is negligible.

## Data Availability

Data are contained within the article.

## Nomenclature

(*x**, *y**, *z**)	dimensionless cartesian orthonormal coordinate system
(*x*, *y*)	cartesian orthonormal coordinate system
ℜ {}	the real part of the periodic EOF function
Re_Ω_	oscillatory Reynolds number
*De*	deborah number *De* = *λ*_1_*ω*
*K*	dimensionless electrokinetic width *K* = *κh*
*z_ν_*	valence of ion
*H*	average half-height of microchannel
*t*	time
*w*(*x*, *y*, *t*)	dimensionless velocity along the z-axis direction
*w_j_*	dimensionless velocity of *δ^j^*-order equation (*j* = 0, 1, 2)
*P*	pressure of the liquid
*G*	dimensionless pressure gradient
*E_0_* *(*t*)	AC electric field
*n_v_*	valence of ion
*n*_0_	ion density of bulk liquid
*e*	elementary charge
*k_b_*	Boltzmann’s constant
*T*	absolute temperature
*U_eo_*	Helmholtz–Smoluchowski electroosmotic velocity
$\bar{u}$	average velocity in rough microchannel
|*U_m_*|	complex amplitude of ac EOF mean velocity
**U**	velocity vector
$u_{0m}$	average velocity in smooth microchannel
$u_{2m}$	increment of average velocity in rough microchannels
Greek symbols	
*α_j_*, *β_j_*	real numbers (*j* = 0, 1, 2)
*λ*	wave number
*θ*	phase difference
*ε*_0_	dielectric constant in vacuum
*λ*_1_	relaxation time or relaxation time of a fluid
***τ***	shear stress tensor
*ρ_e_*	net charge density per unit volume
*η*_0_	zero shear viscosity of fluid
*ω*	oscillation angular frequency of AC electric field
Ψ(*x**, *y**)	electric potential
φ(*x*, *y*)	dimensionless electric potential
φ*_j_*	dimensionless electric potential of δ*^j^* order equation (*j* = 0, 1, 2)
*χ*	phase lag
*ε*	dielectric constant
*δ*	the ratio of the ripple amplitude to the average half-height of the channel
1/*κ*, *λ_D_*	thickness of EDL
*ρ*	density of fluid

## Appendix A

A0=1+ζ2coshK, B0=1−ζ2sinhK, a0=K2(1+iDe)A0(α0+iβ0)2−K2, b0=K2(1+iDe)B0(α0+iβ0)2−K2, C0=−iReΩa0coshK+GiReΩcosh(α0+iβ0), D0=−b0sinhKsinh(α0+iβ0), A1=f1(−1)+f1(1)2cosh(K1), B1=f11−f1(−1)2sinh(K1), A2=g1(−1)2cosh(K1), B2=−g1−12sinh(K1), aj=K2(1+iDe)Aj(α0+iβ0)2−K2, bj=K2(1+iDe)Bj(α0+iβ0)2−K2, (j=1, 2, 3, 4, 5), C1=F1(−1)+F1(1)−2a1cosh(K1)2cosh(α1+iβ1), D1=F11−F1(−1)−2b1sinh(K1)2sinh(α1+iβ1), C2=G1(−1)−2a2cosh(K1)2cosh(α1+iβ1), D2=−G1−1+2b2sinh(K1)2sinh(α1+iβ1),A3=h(−1)+h(1)2cosh(K), B3=h1−h(−1)2sinh(K), A4=f2−1+f212coshK2, B4=f21−f2−12sinhK2, A5=g2(−1)+g2(1)2cosh(K2),B5=g21−g2(−1)2sinh(K2), C3=H(−1)+H(1)−2a3cosh(K)2cosh(α0+iβ0), D3=H1−H(−1)−2b3sinh(K)2sinh(α0+iβ0),C4=F2(−1)+F2(1)−2a4cosh(K2)2cosh(α2+iβ2), D4=F21−F2(−1)−2b4sinh(K2)2sinh(α2+iβ2),C5=G2−1+G21−2a5coshK22coshα2+iβ2, D5=G21−G2−1−2b5sinhK22sinhα2+iβ2.

where, $K_{1}^{2}=\lambda^{2}+K^{2},\;{\left(\alpha_{1}+i\beta_{1}\right)}^{2}=\lambda^{2}+{\left(\alpha_{0}+i\beta_{0}\right)}^{2},$ $f_{1}(-1)=-\cos\theta {\varphi^{\prime }}_{0}(-1),\;f_{1}(1)=-{\varphi^{\prime }}_{0}(1),$ $g_{1}\left(-1\right)=-\sin\theta {\varphi^{\prime }}_{0}(-1),$ $F_{1}(-1)=-\cos\theta {w^{\prime }}_{0}(-1),\;F_{1}(1)=-{w^{\prime }}_{0}(1),$ $G_{1}\left(-1\right)=-\sin\theta {w^{\prime }}_{0}(-1),$ $K_{2}^{2}=4\lambda^{2}+K^{2},\;{\left(\alpha_{2}+i\beta_{2}\right)}^{2}=4\lambda^{2}+{\left(\alpha_{0}+i\beta_{0}\right)}^{2},$ $h\left(-1\right)=-\frac{1}{4}{\varphi^{\prime \prime }}_{0}\left(-1\right)-\frac{1}{2}\cos\theta {f^{\prime }}_{1}\left(-1\right)-\frac{1}{2}\sin\theta {g^{\prime }}_{1}\left(-1\right),$ $h(1)=-\frac{1}{4}{\varphi^{\prime \prime }}_{0}\left(1\right)-\frac{1}{2}{f^{\prime }}_{1}\left(1\right),\;f_{2}\left(1\right)=-\frac{1}{2}{g^{\prime }}_{1}\left(1\right),$ $f_{2}\left(-1\right)=-\frac{1}{4}{\varphi^{\prime \prime }}_{0}\left(-1\right)\sin2\theta -\frac{1}{2}{f^{\prime }}_{1}\left(-1\right)\sin\theta -\frac{1}{2}{g^{\prime }}_{1}\left(-1\right)\cos\theta ,$ $g_{2}\left(-1\right)=\frac{1}{4}{\varphi^{\prime }}_{0}\left(-1\right)\cos2\theta -\frac{1}{2}{g^{\prime }}_{1}\left(-1\right)\sin\theta +\frac{1}{2}{f^{\prime }}_{1}\left(-1\right)\cos\theta ,$ $g_{2}(1)=\frac{1}{4}{\varphi^{\prime \prime }}_{0}\left(1\right)+\frac{1}{2}{f_{1}}^{\prime }\left(1\right),\;H(1)=-\frac{1}{4}{w^{\prime \prime }}_{0}\left(1\right)-\frac{1}{2}{F^{\prime }}_{1}\left(1\right),$ $H\left(-1\right)=-\frac{1}{4}{w^{\prime \prime }}_{0}\left(-1\right)-\frac{1}{2}\cos\theta {F^{\prime }}_{1}\left(-1\right)-\frac{1}{2}\sin\theta {G^{\prime }}_{1}\left(-1\right),$ $F_{2}\left(-1\right)=-\frac{1}{4}{w^{\prime \prime }}_{0}\left(-1\right)\sin2\theta -\frac{1}{2}{F^{\prime }}_{1}\left(-1\right)\sin\theta -\frac{1}{2}{G^{\prime }}_{1}\left(-1\right)\cos\theta ,$ $F_{2}\left(1\right)=-\frac{1}{2}{G^{\prime }}_{1}\left(1\right),\;G_{2}\left(1\right)=\frac{1}{4}{w^{\prime \prime }}_{0}\left(1\right)+\frac{1}{2}{F^{\prime }}_{1}\left(1\right),$ $G_{2}\left(-1\right)=\frac{1}{4}{w^{\prime \prime }}_{0}\left(-1\right)\cos2\theta -\frac{1}{2}{G^{\prime }}_{1}\left(-1\right)\sin\theta +\frac{1}{2}{F^{\prime }}_{1}\left(-1\right)\cos\theta .$
